# Polydatin in respiratory diseases: multi-target mechanisms and therapeutic potential

**DOI:** 10.3389/fphar.2026.1752467

**Published:** 2026-02-13

**Authors:** Chanjun Wan, Xinyang Liu, Yueqi Xu, Li Kang, Xuelong Yu, Min Wang, Min Zhao, Xinhan Li, Zhengtao Chen, Jianguang Wu, Liangji Liu, Xuemei Xu

**Affiliations:** 1 Jiangxi University of Chinese Medicine, Nanchang, Jiangxi, China; 2 Affiliated Hospital of Jiangxi University of Chinese Medicine, Nanchang, Jiangxi, China; 3 Jiangxi Province Key Laboratory of Respiratory Diseases of Chinese Medicine, Nanchang, Jiangxi, China

**Keywords:** molecular targets, pharmacological mechanism, polydatin, respiratory diseases, signaling pathways

## Abstract

Respiratory diseases constitute a heterogeneous group of disorders that primarily involve the lungs. Driven by worsening air pollution, tobacco use, occupational exposures, the COVID-19 pandemic, and population aging, they show persistently high incidence with rising mortality and disability, posing a major global public-health challenge. Current pharmacotherapies—principally antibiotics, glucocorticoids, β_2_-adrenoceptor agonists, and antiviral agents—yield only limited benefit and are constrained by adverse reactions such as gastrointestinal disturbances and hepatorenal toxicity, alongside the escalating problem of drug resistance. The development of safer and more effective therapeutics is therefore of considerable clinical and socioeconomic importance. Plant-derived natural products have attracted increasing interest in the management of respiratory diseases. Polydatin (resveratrol-3-O-β-D-glucoside; also known as piceid; PD) is a stilbenoid polyphenol of plant origin that is widely distributed in Polygonum cuspidatum (Japanese knotweed), Polygonum multiflorum, grapes, peanuts, mulberries, blueberries, and rhubarb. Accumulating evidence indicates that PD exerts anti-inflammatory, antioxidant, antimicrobial, immunomodulatory, and metabolic-regulatory activities and shows potential therapeutic value in pulmonary fibrosis, acute lung injury/acute respiratory distress syndrome, pneumonia, lung cancer, and asthma. This review provides a comprehensive synthesis of the multi-target and multi-pathway mechanisms by which PD acts against respiratory diseases, offering a mechanistic rationale and evidence base to support its clinical development.

## 1 Introduction

The respiratory system is central to gas exchange and immune defense; its dysfunction leads to multi-organ disequilibrium and systemic metabolic disturbances (Hogan and Tata, 2019; Mizgerd, 2012). Respiratory diseases—including pulmonary fibrosis, pneumonia, lung cancer, acute lung injury/acute respiratory distress syndrome (ALI/ARDS), and asthma—collectively rank as the third leading cause of death worldwide and pose a substantial threat to human health. Their pathogenesis is multifactorial, involving oxidative stress, inflammatory injury, immune dysregulation, and metabolic derangements (Chen et al., 2018; Racanelli et al., 2018).

Current pharmacotherapies—principally antibiotics, glucocorticoids, β_2_-adrenoceptor agonists, and antiviral agents—can alleviate symptoms to some extent but rarely halt disease progression (Fournier et al., 2022; Miller et al., 2021). Moreover, the adverse effects associated with corticosteroids, the rapid emergence of antibiotic resistance, and the variable efficacy of antivirals constrained by viral evolution and other complex factors constitute major bottlenecks in treatment (Morris, 1994; Oray et al., 2016). Accordingly, there is an urgent need for natural, multitarget, safe, and effective therapeutic strategies for respiratory diseases.

Plant-derived natural compounds, characterized by unique chemical scaffolds, pleiotropic biological activities, and generally low toxicity, offer promising avenues for intervention (Bahri et al., 2017; Timalsina et al., 2021). Polydatin (PD), a stilbenoid polyphenol originally isolated from the rhizome of the traditional medicinal plant Polygonum cuspidatum (Japanese knotweed), is also abundant in Polygonum multiflorum, grapes, peanuts, mulberries, and blueberries. As a plant-origin natural product, PD combines a distinctive chemical structure with diverse biological effects and a favorable safety profile (Timalsina et al., 2021; Ye et al., 2022). In recent years, a growing body of evidence has indicated that PD exerts beneficial effects across multiple respiratory disorders. This review therefore provides a comprehensive analysis of the effects and multi-target mechanisms of polydatin (PD) across major respiratory diseases, including pulmonary fibrosis, acute lung injury/acute respiratory distress syndrome (ALI/ARDS), pneumonia, lung cancer, and asthma. We focus on its pharmacological mechanisms, molecular targets, and pharmacokinetic characteristics, and discuss key challenges and future directions to better inform subsequent pharmacological research and potential clinical translation. In addition to the main text, relevant information is summarized in tabular form to facilitate rapid retrieval by readers.

## 2 Polydatin

Stilbenoid polyphenols are a class of polyphenolic compounds bearing a 1,2-diphenylethylene moiety and a canonical C6-C2-C6 backbone. Extensive substituent variability on the phenyl rings and cis-trans isomerism confer rich structural diversity at the chemical level, whereas the strong electron-donating capacity of phenolic hydroxyl groups, together with favorable steric configurations, underpins notable biological activity. Consequently, stilbenoids possess considerable value in natural-product-based drug discovery and disease prevention (Zhou et al., 2025).

**FIGURE 1:**
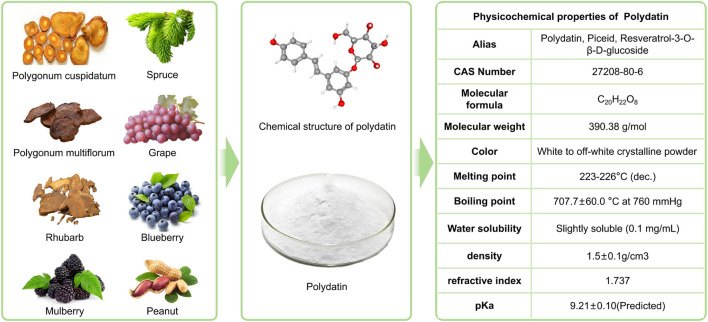
Botanical origin, chemical structure, and physicochemical characteristics of polydatin (PD).

Polydatin (PD), chemically 3,4′,5-trihydroxystilbene-3-O-β-D-glucoside (also known as resveratrol-3-O-β-D-glucoside), is a naturally occurring stilbenoid polyphenol isolated from the rhizome of Polygonum cuspidatum. It possesses the stilbene scaffold (C6-C2-C6), with the molecular formula C_20_H_22_O_8_ and a molecular weight of 390.38 g mol^-1^;The chemical structure of PD, as well as its botanical features and physicochemical characteristics, is illustrated in Figure 1. PD typically appears as a white to off-white crystalline powder with moderate thermal stability. It exhibits poor solubility in water but is soluble in methanol, ethanol, and certain other polar organic solvents (Du et al., 2013). In PD, a glucose moiety is linked to the resveratrol nucleus *via* a glycosidic bond, and hydroxyl groups are present at the 3,4′, and 5 positions of the aglycone. These features collectively underpin multiple pharmacological activities (Karami et al., 2022). The pronounced antioxidant activity of PD is closely related to its multiple phenolic hydroxyls, which can scavenge free radicals and suppress lipid peroxidation. Furthermore, the glycosidic linkage and the specific distribution of hydroxyl groups confer pharmacokinetic advantages, yielding an *in vivo* bioavailability approximately 4-5 fold higher than that of resveratrol and thus overcoming the latter’s rapid metabolism and poor absorption (Liu et al., 2024).

Recent studies highlight the broad health-promoting actions of PD, including anti-inflammatory and anticancer activities; cardioprotective, antidiabetic, gastroprotective, hepatoprotective, and neuroprotective effects (Luo et al., 2022; Wu et al., 2020); benefits in respiratory and renal diseases, rheumatoid disorders, and skeletal health; and protection against multi-organ ischemia-reperfusion injury. With respect to respiratory diseases, PD exhibits multidimensional pharmacological effects—anti-inflammatory, antioxidant, antimicrobial, immunomodulatory, and metabolic-regulatory—indicating substantial translational potential (Shah et al., 2022; Sun and Wang, 2020; Zhang et al., 2024b). However, a systematic and comprehensive synthesis of the current evidence on PD for respiratory indications, particularly its mechanisms of action, remains lacking.

## 3 Methods

### 3.1 Literature search

**FIGURE 2:**
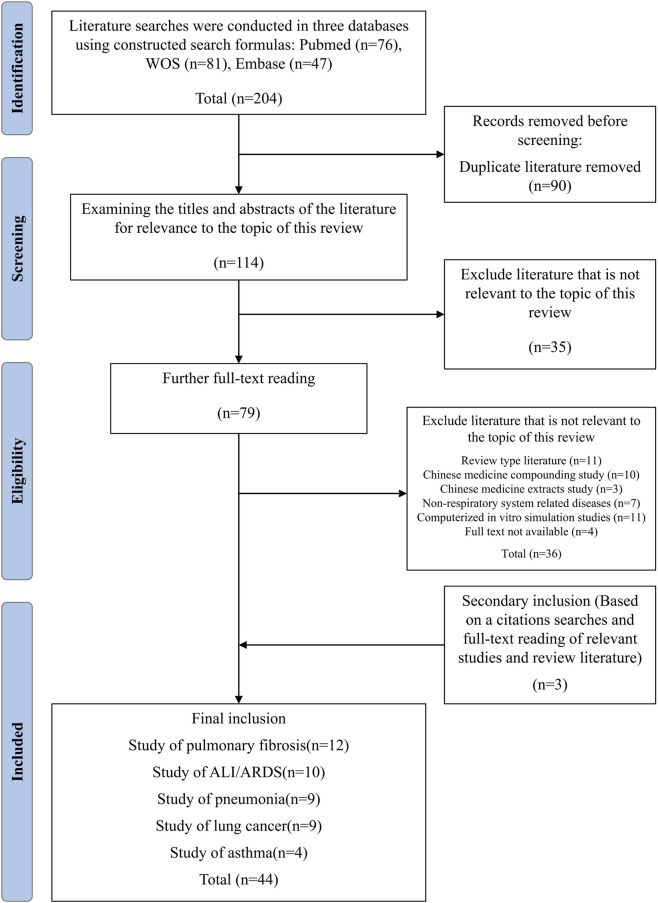
PRISMA-style flow chart of literature identification and selection. Records were retrieved from PubMed, Web of Science, and Embase, followed by removal of duplicates, title and abstract screening, and full-text assessment based on predefined inclusion and exclusion criteria. The numbers of records excluded at each step and the final number of included studies are indicated.

We retrieved literature on the effects of polydatin (PD) against respiratory diseases (RDs) from PubMed, Web of Science, and Embase. Search strings were constructed by appropriately combining keywords and their variants using Boolean operators, including: polydatin, piceid, resveratrol-3-O-glucoside, 3-O-β-D-glucoside (also written 3-O-beta-D-glucoside), 3,4,5-TSG, 3,4,5-trihydroxystilbene-3-β-D-monoglucoside; and disease terms respiratory diseases, respiratory tract diseases, lung diseases, pulmonary fibrosis, acute lung injury, acute respiratory distress syndrome (ARDS), pneumonia, respiratory infection, respiratory tract infections, bacterial pneumonia, viral pneumonia, fungal pneumonia, lung cancer, pulmonary neoplasms, and asthma. The search timeframe spanned from database inception to 30 September 2025. To summarize the mechanisms underlying PD’s interventions in RDs, we screened the title, abstract, and full text of each record. Publications irrelevant to the objectives of this review were excluded. A total of 204 records were initially identified from the databases (PubMed, *n* = 76; Web of Science, *n* = 81; Embase, *n* = 47). After removing duplicates and screening titles/abstracts and full texts according to the predefined criteria, 44 studies were finally included in this review (Figure 2). Included studies were categorized according to the mechanistic actions of PD across different RD categories, with detailed analyses focused on pulmonary fibrosis, acute lung injury/acute respiratory distress syndrome (ALI/ARDS), pneumonia, lung cancer, and asthma.

### 3.2 Study selection and quality assessment

Studies were included if they investigated PD in respiratory disease models. Exclusion criteria included non-English publications, reviews, and studies not involving PD. Study quality and risk of bias were assessed using the SYRCLE tool for animal studies and the Cochrane risk-of-bias tool for clinical studies.

## 4 Effects of polydatin on respiratory diseases and underlying mechanisms

### 4.1 Pulmonary fibrosis

Pulmonary fibrosis is a fatal, chronic interstitial lung disease characterized by fibroblast proliferation, excessive deposition of extracellular matrix (ECM) within the pulmonary interstitium, and remodeling of lung architecture. Its etiology is multifactorial, involving intersecting influences of genetic susceptibility, aging, environmental pollutants, cigarette smoking, occupational exposures, and microbial infections (Koudstaal et al., 2023). The pathobiology of PF encompasses a series of defined cellular programs and molecular pathways, including epithelial injury with aberrant activation, mesenchymal cell activation, immune and inflammatory responses, and dysregulation of profibrotic signaling networks (Moss et al., 2022; Spagnolo et al., 2021). In recent years, the global incidence of PF has continued to rise, imposing substantial healthcare expenditures and precipitating a public health crisis (Gonnelli et al., 2024; Hutchinson et al., 2015). Presently approved antifibrotic agents (pirfenidone and nintedanib) can slow the decline in lung function but neither reverse fibrosis nor halt disease progression, and they are associated with adverse events such as gastrointestinal and dermatologic toxicities as well as potential cardiovascular risks (Galli et al., 2017). Consequently, there is significant socioeconomic impetus to develop safe and effective therapeutics. A growing body of evidence indicates that polydatin confers marked benefits in PF induced by diverse etiologies (Du et al., 2013; Karami et al., 2022; Ye et al., 2022). The underlying mechanisms span anti-inflammatory and antioxidative actions, inhibition of epithelial–mesenchymal transition (EMT), and modulation of the gut microbiota, among others—collectively offering new strategies to overcome current therapeutic bottlenecks.

#### 4.1.1 Modulation of inflammatory networks

Inflammation is a key driver of PF initiation and progression. Chemical agents, airborne pollutants, and pathogens elicit pulmonary inflammation characterized by inflammatory cell infiltration of alveoli, activation of pro-inflammatory signaling pathways, and robust production of mediators such as TNF-α, IL-1β, and IL-6—events integral to fibrogenesis (Racanelli et al., 2018; Wang et al., 2022). Multiple studies demonstrate that PD markedly suppresses activation of inflammatory cascades and the release of pro-inflammatory cytokines, thereby attenuating lung inflammation and mitigating fibrosis. In bleomycin-induced PF rat models, Qiu and colleagues, as well as Yang and colleagues (Qiu et al., 2019; Yang et al., 2023), independently showed that PD targets and inhibits the TNF signaling pathway, reduces alveolitis pathology scores, decreases leukocyte infiltration, collagen deposition, and hydroxyproline accumulation, and downregulates TNF-α, IL-1β, IL-6, and IL-17, thereby alleviating inflammatory injury and slowing fibrotic progression.

These anti-inflammatory effects have also been corroborated *in vitro*. In a paraquat injury model using MRC-5 human embryonic lung fibroblasts, Fu et al. (2020) reported that PD dose-dependently inhibited expression of NLRP3 inflammasome-related proteins (NLRP3, caspase-1, and ASC) and lowered levels of TNF-α, TGF-β, IL-1β, and IL-6, with the most pronounced effect at 100 μmol/L PD. Beyond chemically induced PF, PD also mitigates fibrosis triggered by physical toxicants. In a silica-induced silicosis rat model, Wu et al. (2025) found that PD suppressed TNF signaling and significantly reduced protein and mRNA expression of pro-inflammatory cytokines (TNF-α, IL-6, IL-1β) and apoptosis-related targets (p53, caspase-3), thereby retarding fibrotic progression. Importantly, PD exerts protective effects against pathogen-associated pulmonary inflammation and subsequent fibrosis: in a *Mycoplasma* pneumoniae infection model, Tang et al. (2019) demonstrated that PD dampened aberrant activation of the NLRP3 inflammasome and NF-κB pathways, curtailed inflammatory mediator expression, and alleviated inflammation-related lung injury and fibrosis. Collectively, PD effectively counteracts inflammation-driven fibrogenesis across toxicant- and pathogen-induced settings, largely through modulation of TNF and NF-κB signaling and suppression of pro-inflammatory cytokine release.

#### 4.1.2 Anti-oxidative stress

Physiological redox homeostasis reflects a dynamic balance between oxidant generation and antioxidant defenses. When this balance is disrupted and reactive oxygen species (ROS) accumulate beyond the scavenging capacity of antioxidant systems, oxidative stress ensues, leading to cytotoxicity and tissue damage. Substantial evidence implicates oxidative stress in the pathogenesis of PF and other disorders such as diabetes and cardiovascular disease (Alfadda and Sallam, 2012; Otoupalova et al., 2020). PD enhances antioxidant capacity through multiple pathways, thereby reducing ROS burden, mitigating oxidative injury, and slowing PF progression. In a radon exposure-induced PF model, PD inhibited the PI3K/AKT/mTOR pathway, significantly decreased epithelial ROS levels, increased superoxide dismutase (SOD) activity in cells and mouse serum, and lowered serum malondialdehyde (MDA) (Chen et al., 2023). PD’s antioxidant effects were further validated in bleomycin-challenged Sprague-Dawley rats: Liu et al. (2020) showed that PD targeted the TGF-β1/Smad/ERK axis, elevated SOD activity, and reduced MDA and myeloperoxidase (MPO), with the high-dose PD group (160 mg/kg) achieving efficacy comparable to the reference drug pirfenidone. Additionally, in a model of inhalational exposure to artificially generated PM2.5 (aPM2.5), Yan et al. (2017) found that PD activated the pulmonary Nrf2/PPAR-γ axis, decreased the oxidative potential (OP) of aPM2.5, suppressed ROS production, and increased glutathione peroxidase (GSH-Px) activity, thereby strengthening cellular resilience to oxidative stress, reducing lung injury, and delaying fibrotic development.

#### 4.1.3 Inhibition of epithelial-mesenchymal transition (EMT)

EMT—the conversion of epithelial cells into motile mesenchymal cells—is a pivotal pathological process in PF(Chapman, 2011). PD inhibits EMT *via* several signaling routes, thereby restraining fibrotic progression. Within the TGF-β1/Smad/ERK pathway, PD reduces TGF-β1 expression, lowers Smad2 and Smad3 levels, and diminishes production of type I and III collagen and their procollagen propeptides (PICP and PIIINP). PD also decreases markers associated with matrix deposition and myofibroblast activation (hydroxyproline, α-SMA) while restoring E-cadherin, collectively suppressing EMT. Notably, at high dose (160 mg/kg), PD achieved a 17.5% inhibition of TGF-β1—exceeding the 12.3% observed with pirfenidone in the referenced studies (Liu et al., 2020; Qiu et al., 2019).

Through the PI3K/AKT/mTOR pathway, PD inhibits phosphorylation of PI3K, AKT, and mTOR, downregulates mesenchymal markers (FN1, vimentin), blocks EMT progression, and reduces collagen fiber deposition (Chen et al., 2023; Zhang et al., 2025). PD also coordinates additional pathways to suppress EMT and fibrosis: in an interstitial lung disease (ILD) mouse model, Zhang et al. (2024a) reported that PD activated AMPK/PGC-1α/PPARγ signaling while inhibiting HMGB1/RAGE signaling, thereby reducing aberrant expression of HMGB1, RAGE, SEPTIN4, ACTA2, and ITGAV and jointly repressing EMT and fibrotic responses. Consistently, Zeng et al. (2019) demonstrated that PD promoted Nrf2-mediated antioxidant defenses, decreased ROS and TGF-β1 levels, reversed TGF-β1-induced EMT, and ameliorated pulmonary fibrosis.

#### 4.1.4 Regulation of the lung-gut axis and remodeling of the intestinal microbiota

The onset and progression of PF are closely linked to dysbiosis and metabolic perturbations of the intestinal microbiota (Dong et al., 2024). PD improves PF by modulating the lung-gut axis, reshaping microbial communities, and altering their metabolites. In a bleomycin-induced PF model in C57BL/6 mice, Yang et al. (2023) showed that PD significantly increased the abundance of beneficial taxa (Muribaculaceae, *Lactobacillus*, Akkermansia) and promoted the expansion of probiotic genera such as Dubosiella, thereby remodeling the microbial ecosystem and attenuating PF. In a silica-induced silicosis model in Sprague-Dawley rats, Wu et al. (2025) further demonstrated that PD reversed the aberrant elevation of α diversity, decreased the relative abundance of phyla such as Elusimicrobiota, and increased levels of short-chain fatty acids (SCFAs) including propionate and acetate in colonic contents. SCFAs are key microbial metabolites with immunoregulatory and anti-inflammatory functions; perturbations in the gut microbiota-SCFA network correlate with the severity of lung injury. These findings suggest that PD exerts anti-fibrotic effects, at least in part, by modulating the gut microbiota-immune-metabolic axis.

**FIGURE 3:**
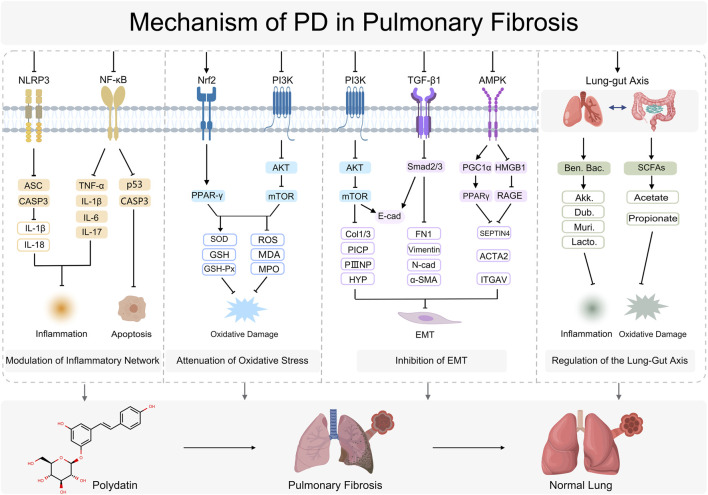
Mechanism of PD in Pulmonary Fibrosis. NOD-, LRR- and pyrin domain-containing protein 3 (NLRP3), Nuclear Factor Kappa-Light-Chain-Enhancer of Activated B Cells (NF-κB), Apoptosis-Associated Speck-like Protein Containing a CARD (ASC), CASP3 (Caspase-3), Interleukin-1 Beta (IL-1β), Interleukin-18 (IL-18), Tumor Necrosis Factor Alpha (TNF-α), Interleukin-6 (IL-6), Interleukin-17 (IL-17), Tumor Protein p53 (p53), Nuclear Factor Erythroid 2-Related Factor 2 (Nrf2), Peroxisome Proliferator-Activated Receptor Gamma (PPAR-γ), Phosphoinositide 3-Kinase (PI3K), Protein Kinase B (AKT), Mechanistic Target of Rapamycin (mTOR), Superoxide Dismutase (SOD), Glutathione (GSH), Glutathione Peroxidase (GSH-Px), Reactive Oxygen Species (ROS), Malondialdehyde (MDA), Myeloperoxidase (MPO), Transforming Growth Factor Beta 1 (TGF-β1), Mothers Against Decapentaplegic Homolog 2/3 (Smad2/3), E-cadherin (E-cadherin), Fibronectin 1 (FN1), Collagen Type I and Type III (Col I/III), Procollagen Type I C-Terminal Propeptide (PICP), Procollagen Type III N-Terminal Propeptide (PIIINP), Hydroxyproline (HYP), Vimentin (Vimentin), N-cadherin (N-cadherin), Alpha-Smooth Muscle Actin (α-SMA), AMP-Activated Protein Kinase (AMPK), Peroxisome Proliferator-Activated Receptor Gamma Coactivator 1-Alpha (PGC1α), High Mobility Group Box 1 (HMGB1), Receptor for Advanced Glycation Endproducts (RAGE), Septin 4 (SEPTIN4), Actin Alpha 2, Smooth Muscle (ACTA2), Integrin Alpha V (ITGAV), Beneficial Bacteria (Ben. Bac), Akkermansia (Akk), Dubosiella (Dub), Muribaculaceae (Muri), *Lactobacillus* (Lacto), Short-Chain Fatty Acids (SCFAs), Acetate (Acetate), Propionate (Propionate).

In summary, PD is a pleiotropic natural compound that combats PF through convergent mechanisms—reprogramming inflammatory networks, counteracting oxidative stress, inhibiting EMT, and remodeling the intestinal microbiota. Across multiple PF models, PD has shown efficacy in preclinical models that is comparable to, and in some instances approaches or exceeds, that of standard-of-care agents in these experimental settings, with a favorable safety profile. These converging lines of evidence underscore PD’s therapeutic value and translational potential in PF (Figure 3).

### 4.2 Acute lung injury/acute respiratory distress syndrome (ALI/ARDS)

Acute lung injury (ALI) is an acute, diffuse, inflammatory injury to the lungs caused by a variety of pulmonary and extrapulmonary insults, primarily damaging alveolar epithelial cells and pulmonary vascular endothelial cells; progression can culminate in acute respiratory distress syndrome (ARDS)(Papazian et al., 2021). ALI and ARDS represent sequential stages along a common disease continuum. Etiologies include pneumonia, extrapulmonary sepsis, aspiration, trauma, and such risk modifiers as cigarette smoking, alcohol misuse, blood transfusion, and prolonged exposure to ozone and other air pollutants (Pisani et al., 2022). Pathologically, widespread epithelial and endothelial injury increases the permeability of the alveolar-capillary barrier, leading to pulmonary edema and gas exchange failure (Ma et al., 2025). Epidemiological data indicate a rising incidence of ARDS, reaching 306 per 100,000 persons per year in individuals aged 75–84 years (Papazian et al., 2021); in-hospital mortality remains high at 34.9%, 40.3%, and 46.1% for mild, moderate, and severe ARDS, respectively (Bellani et al., 2016). Mechanical ventilation remains the cornerstone of supportive care, while emerging approaches—such as cell-based therapies with mesenchymal stromal cells (MSCs)(De Luca et al., 2020; Yip et al., 2020) and immuno-inflammatory pathway modulation (e.g., the IL-1 receptor antagonist anakinra)—are under active investigation. Substantial evidence shows that polydatin (PD) exerts multidimensional protective effects in ALI/ARDS through anti-inflammatory and antioxidant actions, inhibition of apoptosis, and activation of mitophagy, offering new directions for therapy.

#### 4.2.1 Inhibition of core inflammatory signaling

Runaway inflammation is the central pathophysiological driver of ALI/ARDS; excessive activation of inflammatory cascades precipitates diffuse lung injury and respiratory failure (Bos and Ware, 2022). Across diverse ALI models, PD’s anti-inflammatory protection has been validated and linked to modulation of key signaling pathways. In a lipopolysaccharide (LPS)-induced rat ALI model, Li et al. (2014a) showed that PD significantly reduced levels of pro-inflammatory mediators—including TNF-α, IL-6, IL-1β, and high mobility group box 1 (HMGB1)—in bronchoalveolar lavage fluid (BALF) and serum, decreased myeloperoxidase (MPO) activity in lung tissue, and lowered neutrophil counts and protein content in BALF, thereby attenuating inflammatory lung injury (Li et al., 2014a; Li et al., 2014b). In the LPS-induced ALI model in BALB/c mice and BEAS-2B human bronchial epithelial cells, Jiang et al. further demonstrated that PD dose-dependently downregulated IL-1β, IL-6, IL-8, and TNF-α. Mechanistically, PD interrupted TLR4-MyD88-NF-κB signaling by downregulating Toll-like receptor 4 (TLR4) and its adaptor myeloid differentiation primary response protein 88 (MyD88), suppressing phosphorylation of IκB kinase (IKK) and IκBα, and thereby preventing NF-κB nuclear translocation and transcriptional activation (Jiang et al., 2015). In a cecal ligation and puncture (CLP) sepsis-induced ALI model, Liao et al. confirmed PD’s suppression of these cytokines *in vivo*, and complementary *in vitro* data implicated downregulation of the transcription factor Spi-B as an upstream mechanism by which PD restrains activation of the PI3K/AKT and NF-κB pathways (Liao et al., 2023).

#### 4.2.2 Activation of endogenous antioxidant defenses

An imbalance between oxidant production and antioxidant defenses is a second major contributor to lung injury, amplifying oxidative stress and pathological damage (Wang et al., 2025). PD exhibits robust control over redox homeostasis by activating endogenous antioxidant systems. In multiple models—including LPS-induced ALI, radiation injury, and radon exposure—PD significantly decreased lipid peroxidation products (LPO), malondialdehyde (MDA), and intracellular reactive oxygen species (ROS), while increasing superoxide dismutase (SOD) activity, thereby restoring the oxidant-antioxidant balance and limiting oxidative damage. In BEAS-2B cells and a murine thoracic irradiation model, Cao et al. (2017) found that PD activated the Sirt3-Nrf2/PGC-1α regulatory axis, augmenting pulmonary antioxidant defenses at the pathway level. In a radon exposure model, Chen et al. reported that PD downregulated phosphorylated AKT and mTOR in radon-exposed bronchial epithelial cells (BEAS-2B, 16HBE) and reduced phosphorylated PI3K in lung tissue of exposed mice; by suppressing aberrant PI3K/AKT/mTOR activation, PD curtailed upstream drivers of persistent oxidative stress (Chen et al., 2023). Moreover, in LPS-challenged RAW 264.7 cells and a *Pseudomonas aeruginosa* PA14 infection model, Chi et al. showed that PD directly bound the DLG motif of Nrf2, disrupted the KEAP1-Nrf2 interaction, inhibited Nrf2 ubiquitination and degradation, and promoted Nrf2 nuclear translocation, thereby inducing antioxidant response element (ARE)-regulated genes such as HO-1 and GCLC and strengthening global cellular antioxidant capacity (Chi et al., 2024). Together, these findings indicate that PD engages multiple nodes—Sirt3-Nrf2/PGC-1α, Nrf2, and PI3K/AKT/mTOR—to enhance resistance to oxidative stress in the injured lung.

#### 4.2.3 Regulation of apoptosis

Excessive apoptosis of alveolar epithelial and endothelial cells is pivotal to alveolar-capillary barrier failure (Gregoire et al., 2018). PD mitigates apoptosis by modulating Bcl-2 family proteins to stabilize the mitochondrial membrane. In burn- and LPS-induced rat ALI models, PD markedly upregulated anti-apoptotic Bcl-2 and Bcl-xL while downregulating pro-apoptotic Bax; this shift reduced mitochondrial release of cytochrome c (CYCS), suppressed activation of caspase-3, and significantly decreased the number of TUNEL-positive apoptotic cells, thereby preserving lung structural integrity (Li et al., 2014a; Li et al., 2014b). Complementing its anti-oxidative actions, PD also curtailed apoptosis in radon-exposed ALI cells by blocking PI3K/AKT/mTOR signaling (Chen et al., 2023). In LPS-induced ARDS models *in vivo* and *in vitro*, Li et al. (2019) further showed that PD promoted translocation of the E3 ubiquitin ligase Parkin from the cytosol to damaged mitochondria, activating mitophagy to clear dysfunctional organelles, dampen mitochondria-dependent apoptotic signaling, and enhance cellular resilience to injury.

#### 4.2.4 Additional mechanisms

Beyond anti-inflammatory, antioxidant, and anti-apoptotic effects, PD exerts additional protective actions in ALI. Shu et al. reported that PD dose-dependently increased the expression of club cell secretory protein (CCSP; also known as SCGB1A1) in lung tissue and bronchial epithelial cells and inhibited phospholipase A2 (PLA2) activity. These changes reduced degradation of pulmonary surfactant and formation of inflammatory lipid mediators, ultimately lowering pulmonary microvascular permeability and alleviating edema (Shiyu et al., 2011). In a mouse model of traumatic brain injury (TBI)-associated ALI, Gu et al. (2021) found that PD downregulated the damage-associated molecular pattern (DAMP) molecule S100B, thereby suppressing formation of neutrophil extracellular traps (NETs), mitigating NET-driven hyperinflammation and vascular leak. This mechanism interrupts a key pathogenic link along the brain-lung axis and provides a molecular basis for PD’s efficacy in ALI secondary to neurotrauma.

**FIGURE 4:**
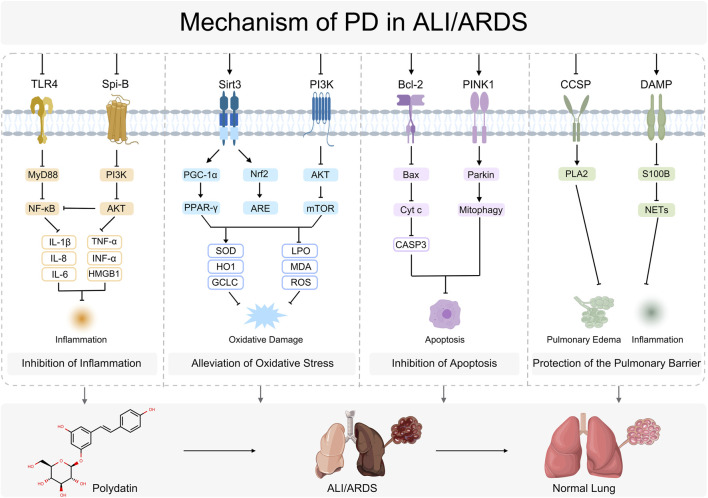
Mechanism of PD in ALI/ARDS.Toll-like receptor 4 (TLR4), Myeloid differentiation primary response 88 (MyD88), Nuclear factor kappa-light-chain-enhancer of activated B cells (NF-κB), Interleukin-1 beta (IL-1β), Interleukin-8 (IL-8), Interleukin-6 (IL-6), Spi-B transcription factor (Spi-B), Phosphoinositide 3-kinase (PI3K), PPARγ coactivator 1-alpha (PGC-1α), AKT serine/threonine kinase (AKT), Tumor necrosis factor alpha (TNF-α), Interferon alpha (IFN-α), High mobility group box 1 (HMGB1), Sirtuin 3 (Sirt3), Nuclear factor erythroid 2-related factor 2 (Nrf2), Peroxisome proliferator-activated receptor gamma (PPAR-γ), Antioxidant response element (ARE), Superoxide dismutase (SOD), Heme oxygenase 1 (HO-1), Glutamate-cysteine ligase catalytic subunit (GCLC), B-cell lymphoma 2 (Bcl-2), BCL2-associated X protein (Bax), Mechanistic target of rapamycin (mTOR), Cytochrome c (Cyt c), Lipid peroxidation (LPO), Malondialdehyde (MDA), Reactive oxygen species (ROS), PTEN induced putative kinase 1 (PINK1), Parkin RBR E3 ubiquitin protein ligase (Parkin), Caspase-3 (CASP3), Mitophagy (Mitophagy), Club cell secretory protein (CCSP), Phospholipase A2 (PLA2), S100 calcium binding protein B (S100B), Damage-associated molecular pattern (DAMP), Neutrophil extracellular traps (NETs).

In sum, current evidence delineates PD as a multitarget natural compound capable of systemically recalibrating the complex pathophysiological networks underpinning ALI/ARDS. Through concurrent modulation of inflammatory signaling, reinforcement of endogenous antioxidant defenses, suppression of apoptosis with promotion of mitophagy, and additional barrier protective effects, PD shows substantial promise for the prevention and treatment of ALI/ARDS (Figure 4).

### 4.3 Pneumonia

Pneumonia is an infectious disease of the respiratory tract caused by pathogenic microorganisms or physicochemical insults, characterized pathologically by inflammatory injury of the alveoli, pulmonary interstitium, and bronchioles. The World Health Organization estimates ∼450 million cases annually, with >4 million deaths worldwide, making pneumonia a leading cause of morbidity and mortality. The global disease burden of pneumonia surpasses that of major diseases such as cancer, diabetes, and HIV/AIDS (Mizgerd, 2012). In the United States alone, the annu (Mizgerd, 2012) community-acquired pneumonia (CAP) exceeds US$17 billion (File and Marrie, 2010). Pneumonia is primarily precipitated by microbial infection but is also driven by the host response, engaging multiple physiological systems (Quinton et al., 2018). A wide array of pathogens can cause pneumonia, including respiratory viruses, bacteria, fungi, and *Mycoplasma* spp. Viruses and *Mycoplasma* account for 40%–60% of CAP, whereas bacteria (e.g., *Staphylococcus aureus*) are the principal pathogens in hospital-acquired pneumonia (HAP). Approximately 200 million cases of viral CAP occur each year (Ruuskanen et al., 2011). Current therapy relies mainly on antimicrobial agents and supportive care; however, antimicrobial resistance, viral variation, and multisystem complications pose persistent therapeutic bottlenecks (Prina et al., 2015), underscoring the need for novel interventions.

In recent years, PD and its combination formulations have demonstrated dual anti-pathogen and immunomodulatory activities. Mechanistically, PD suppresses pathogen replication, modulates inflammatory signaling pathways, and attenuates pyroptosis and oxidative-stress injury. Across viral, bacterial, and mycoplasmal pneumonia, PD has shown substantial translational potential, offering new avenues to overcome current therapeutic limitations.

#### 4.3.1 Viral pneumonia

The core pathogenesis of viral pneumonia involves robust intrahost viral replication following cell entry, accompanied by excessive inflammation and programmed cell death, culminating in lung injury. PD exerts both direct antiviral effects and immune modulation, achieving synergistic antiviral and anti-inflammatory outcomes. Xu et al. (2021) identified PD as a putative broad-spectrum coronavirus inhibitor that effectively suppresses the proteolytic activities of SARS-CoV-2 and MERS-CoV 3-chymotrypsin-like protease (3CLpro)/main protease (Mpro) and papain-like protease (PLpro), thereby blocking viral replication. De Angelis et al. (2021) further showed that PD interferes with the viral transcription-replication program to inhibit viral protein synthesis, conferring marked antiviral and anti-inflammatory effects against influenza A virus and SARS-CoV-2 *in vitro*. In addition, Sun et al. (2022) elucidated PD’s protection against virus-induced cellular injury: SARS-CoV-2 nonstructural protein NSP6 binds ATP6AP1, impairing lysosomal acidification and arresting autophagic flux, which activates the NLRP3/ASC-dependent caspase-1 pathway, triggers pyroptosis of pulmonary epithelial cells, and promotes IL-1β/IL-18 release. PD targets this cascade by restoring autophagolysosomal function, suppressing pyroptosis and hyperinflammation, and thereby mitigating virus-mediated tissue damage.

#### 4.3.2 Bacterial pneumonia

In bacterial pneumonia, PD primarily acts by reprogramming inflammatory signaling and oxidative-stress responses, dampening the excessive host inflammation elicited by bacterial components and reducing parenchymal injury. *S. aureus* is a common HAP pathogen; its cell-wall lipoteichoic acid (LTA) is a key virulence factor. Using RAW 264.7 macrophages and BALB/c mice, Zhao et al. (2017) showed that LTA activates Toll-like receptor 2 (TLR2) to drive NF-κB signaling, resulting in overproduction of pro-inflammatory mediators (TNF-α, IL-1β, IL-6). PD attenuated this response in a dose-dependent manner by inhibiting the TLR2/MyD88/NF-κB axis and reducing nuclear translocation of NF-κB p65, thereby markedly suppressing LTA-induced inflammation. Zhao et al. further found that LTA induces excessive intracellular reactive oxygen species (ROS) and activates the caspase-9/3 cascade, leading to apoptosis. PD pretreatment lowered LTA-induced ROS, restrained NF-κB nuclear translocation, and reduced caspase-9/3 activation and apoptosis. Collectively, PD combats bacterial pneumonia *via* dual modulation of inflammatory pathways and oxidative stress.

#### 4.3.3 Mycoplasmal pneumonia

*Mycoplasma* spp. lack a cell wall and use adhesins to bind host cell-surface receptors, activating inflammatory signaling and inducing cellular injury; chronic or unresolved infection may promote pulmonary fibrosis. PD targets key pathogenic pathways of *Mycoplasma*, suppressing infection-induced inflammation and pyroptosis while helping prevent subsequent fibrotic remodeling. In BEAS-2B cells and BALB/c mice, Chen et al. (2023) showed that *Mycoplasma* pneumoniae (MP) activates caspase-1, generating the N-terminal fragment of gasdermin D (GSDMD-N) that forms membrane pores, thereby causing pyroptosis and robust release of IL-1β, IL-18, and lactate dehydrogenase (LDH). PD inhibited this caspase-1/GSDMD pathway in a dose-dependent fashion, reducing GSDMD-N formation and cytokine release, and significantly alleviating MP-induced epithelial pyroptosis and lung injury *in vitro* and *in vivo*. Given the long-term risk of fibrosis, Tang et al. combined *in vivo* and *in vitro* analyses and found that PD protects against MP pneumonia (MPP) by suppressing activation of the NLRP3 inflammasome (NLR family pyrin domain-containing 3) and the nuclear factor-κB (NF-κB) pathway, thereby constraining post-infection pulmonary inflammation and fibrotic progression. Consistent benefits were also observed in *Mycoplasma* gallisepticum (MG) models. Zou et al. (2020) demonstrated that PD dose-dependently inhibited MG growth *in vitro*, restored MG-suppressed viability of DF-1 cells, and reversed MG-induced G1 cell-cycle arrest and apoptosis. *In vivo*, PD ameliorated MG-driven inflammatory cell infiltration, epithelial desquamation, and interstitial congestion in embryonic chicken lungs, while inhibiting TLR6/MyD88/NF-κB activation and downstream cytokine secretion. Notably, its efficacy was comparable to that of the macrolide antibiotic tylosin.

**FIGURE 5:**
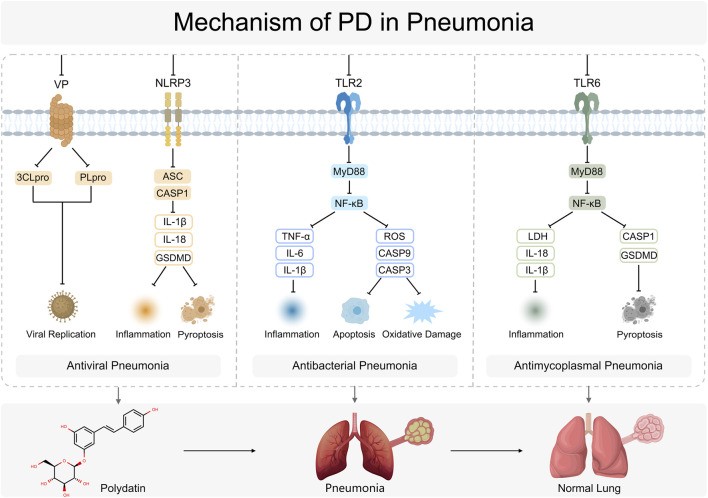
Mechanism of PD in Pneumonia.NOD-, LRR- and pyrin domain-containing protein 3 (NLRP3), Toll-like receptor 2 (TLR2), 3C-like protease (3CLpro), Papain-like protease (PLpro), Apoptosis-associated speck-like protein containing a CARD (ASC), Caspase-1 (CASP1), Interleukin-18 (IL-18), Gasdermin D (GSDMD), Myeloid differentiation primary response 88 (MyD88), Nuclear factor kappa-light-chain-enhancer of activated B cells (NF-κB), Tumor necrosis factor alpha (TNF-α), Interleukin-6 (IL-6), Reactive oxygen species (ROS), Caspase-9 (CASP9), Caspase-3 (CASP3), Lactate dehydrogenase (LDH).

In sum, PD exhibits favorable therapeutic effects against pneumonia caused by viruses, bacteria, and *Mycoplasma* by virtue of its multi-target profile. Its additional advantages-low toxicity, broad-spectrum activity, and a promising resistance profile-position PD as a compelling candidate for pneumonia therapy and a potential adjunct to existing antimicrobial regimens (Figure 5).

### 4.4 Lung cancer

Lung cancer (LC) is a malignant tumor arising from the bronchial mucosal epithelium, bronchial glands, or alveolar epithelium and remains one of the leading causes of cancer incidence and mortality worldwide. According to the International Agency for Research on Cancer (IARC), there were 2.5 million new LC cases in 2022, accounting for 12.4% of all newly diagnosed cancers (Smolarz et al., 2025). Lung carcinogenesis is a complex, multistep, multifactorial, and multigenic process in which oncogenic exposures drive genomic mutations and dysregulated signaling, culminating in uncontrolled proliferation, invasion, and metastasis (Su et al., 2020). Although substantial progress has been achieved with surgery, radiotherapy, chemotherapy, targeted therapy, and immunotherapy, major challenges persist, including narrow eligibility, acquired resistance, and dose-limiting toxicities. A growing body of evidence indicates that polydatin (PD) exerts robust antitumor activity—both as monotherapy and in combination regimens—by suppressing proliferation, inducing apoptosis, and remodeling the tumor microenvironment (TME), with notable chemosensitizing and radiosensitizing effects that underscore its translational promise.

#### 4.4.1 Inhibition of tumor proliferation and induction of apoptosis

Suppressing cancer-cell proliferation and triggering programmed cell death are central mechanisms of PD’s anti-LC activity. In a concentration-dependent manner, PD markedly reduces the viability of multiple non-small cell lung cancer (NSCLC) cell lines, including A549 and NCI-H1975. In A549 cells, PD induces DNA damage and cellular senescence and ultimately apoptosis, thereby exerting antitumor effects (Verma and Tiku, 2022). Consistently, PD inhibits A549 and NCI-H1975 proliferation in a dose- and time-dependent fashion, induces apoptosis, and causes S-phase arrest, thereby preventing cell-cycle progression (Zhang et al., 2014). Complementing these findings, *in vitro* and *in vivo* studies show that PD suppresses the invasive and proliferative capacities of Lewis lung carcinoma (LLC; a murine NSCLC model) and downregulates epidermal growth factor receptor (EGFR) and tumor necrosis factor (TNF) protein expression, collectively restraining NSCLC tumor growth (Fan and Shuai, 2024).

#### 4.4.2 Modulation of the tumor microenvironment and antitumor immunity

Beyond direct cytotoxicity, PD modulates the TME and antitumor immune responses. Inflammation and immune suppression within the TME are key drivers of lung cancer progression. PD attenuates these processes by inhibiting pro-inflammatory signaling and lowering cytokine expression. Given the pivotal role of NF-κB activation in malignant progression, studies using human LC cell lines A549 and H1299 demonstrate that PD suppresses NF-κB signaling in a dose-dependent manner, downregulates NLRP3 inflammasome activation, and reduces the release of IL-1β and IL-18. This reprograms the immunosuppressive TME and curtails proliferation, migration, and invasion of A549 and H1299 cells (Zou et al., 2018). These observations suggest that PD not only directly restrains tumor cells but may also potentiate antitumor immunity, providing a mechanistic rationale for combination with immunotherapy.

### 4.5 Complementary sensitization of standard therapies

While targeted agents offer precision and efficacy, long-term use commonly engenders resistance and can elicit cutaneous and gastrointestinal toxicities (Meert and Cadranel, 2015); radiotherapy (RT) achieves high local control in early-stage disease but is limited by radioresistance and collateral injury to adjacent normal tissues (Vinod and Hau, 2020). PD complements these modalities by enhancing efficacy, mitigating resistance, and reducing adverse effects.

#### 4.5.1 Chemotherapy combinations

In NSCLC models, PD enhances cisplatin-induced apoptosis by activating reactive oxygen species (ROS)-mediated endoplasmic reticulum (ER) stress and the JNK/p38 MAPK pathway (Wu et al., 2024). This effect requires PD-driven upregulation of NADPH oxidase 5 (NOX5); NOX5 knockout attenuates PD-mediated ROS cytotoxicity, and xenograft studies corroborate the combinatorial antitumor efficacy.

#### 4.5.2 Management of targeted-therapy skin toxicity

In a prospective pilot study, prophylactic topical 1.5% polydatin cream reduced the incidence of afatinib-associated rash to 41.2%, with no grade-3 events and no treatment discontinuations due to dermatologic toxicity. Efficacy was comparable to tetracyclines and was achieved without additional adverse effects (Fuggetta et al., 2019). These data suggest that PD—via modulation of the EGFR axis and local anti-inflammatory activity—can alleviate EGFR-inhibitor skin toxicity and improve patients’ quality of life.

#### 4.5.3 Radiotherapy combinations

PD functions as a radiosensitizer in lung cancer, enhancing tumor radiosensitivity while attenuating RT-induced B-cell infiltration and inflammatory responses, thereby reducing normal-tissue injury (Guo et al., 2023). In animal models, PD plus RT significantly decreases tumor volume with concomitantly less collateral tissue damage, indicating improved therapeutic index and favorable safety.

**FIGURE 6:**
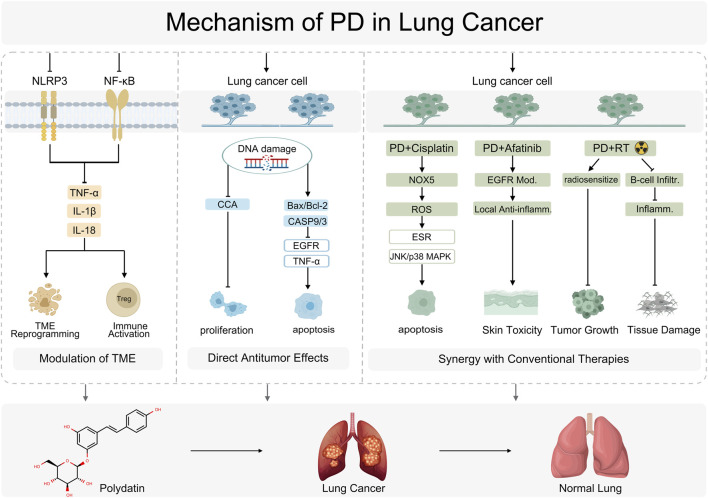
Mechanism of PD in Lung Cancer.NOD-, LRR- and pyrin domain-containing protein 3 (NLRP3), Nuclear factor kappa-light-chain-enhancer of activated B cells (NF-κB), Tumor necrosis factor alpha (TNF-α), Interleukin-1 beta (IL-1β), Cholangiocarcinoma (CCA), BCL2-associated X protein (Bax), B-cell lymphoma 2 (Bcl-2), Caspase-9 (CASP9), Caspase-3 (CASP3), Epidermal growth factor receptor (EGFR), Pembrolizumab plus Cisplatin (PD + Cisplatin), NADPH oxidase 6 (NOX6), Reactive oxygen species (ROS), Estrogen receptor (ESR), c-Jun N-terminal kinase (JNK), p38 mitogen-activated protein kinase (p38 MAPK), B-cell infiltration (B-cell infiltr.), Pembrolizumab plus Afatinib (PD + Afatinib), Epidermal growth factor receptor modulation (EGFR Mod.), Local Anti-inflammatory (Local Anti-inflamm.), Inflammation (Inflamm.).

In sum, PD’s antitumor efficacy against lung cancer is supported by convergent evidence: it suppresses proliferation, induces apoptosis, and reconditions the TME, while synergizing with chemotherapy, targeted therapy, and RT. Nevertheless, poor aqueous solubility and low oral bioavailability remain barriers to clinical translation. Formulation and delivery innovations are addressing these limitations. For example, cocrystallization of PD with L-proline (PD-L-Pro) increased solubility and dissolution rate by approximately 15.8% and preserved potent cytotoxicity against A549 cells while significantly reducing toxicity toward normal HEK-293 cells (Lou et al., 2021). In parallel, nanodelivery systems have been engineered to enhance targeting and exposure: layer-by-layer-assembled, hyaluronic acid/lactoferrin dual-coated, PD-loaded PLGA nanoparticles actively target CD44 receptors on lung cancer cells, improve cellular uptake, and augment antitumor activity, while also enhancing PD’s solubility and stability (Nashaat et al., 2024). Collectively, these strategies mitigate pharmacokinetic liabilities and improve specificity and safety, charting a feasible path from bench to bedside and broadening the therapeutic horizon for PD in lung cancer (Figure 6).

### 4.6 Asthma

Asthma is a chronic inflammatory airway disorder with multifactorial etiology encompassing genetic susceptibility, environmental exposures, and lifestyle factors. Airway hyperresponsiveness (AHR) and airway remodeling are its cardinal pathological features. The global prevalence of asthma continues to rise. Current management relies primarily on bronchodilators and glucocorticoids; however, limitations include drug dependence, adverse effects, and suboptimal efficacy in severe disease. Recent studies indicate that polydatin (PD) can modulate asthma pathogenesis through complementary mechanisms—including anti-inflammatory and antioxidant effects, inhibition of airway remodeling, and regulation of iron metabolism—thereby offering a potential avenue for safer and more effective therapy.

#### 4.6.1 Anti-inflammatory and antioxidant actions

PD exhibits pronounced anti-inflammatory and antioxidant activities and has shown substantial therapeutic potential in asthma models, with mechanisms validated across multiple experimental dimensions.

#### 4.6.2 Anti-inflammatory effects

In an asthmatic mouse model, Hanna et al. (2019) reported that PD alleviated airway inflammation by downregulating pulmonary surfactant protein-D (SP-D) and urocortin (UCN). PD also significantly reduced serum immunoglobulin E (IgE) and decreased the expression of key cytokines (IL-4, IL-5, IL-13, IFN-γ, and TNF-α). Concomitantly, PD restored redox balance—lowering malondialdehyde (MDA) while elevating glutathione (GSH) and superoxide dismutase (SOD) activities. Histopathological assessment corroborated these findings: PD improved airway architecture and reduced inflammatory cell infiltration, with efficacy comparable to dexamethasone.

#### 4.6.3 Antioxidant effects *via* Nrf2 signaling

Using *in vitro* and *in vivo* approaches, Zeng et al. (2019) demonstrated that PD activates the nuclear factor erythroid 2-related factor 2 (Nrf2) pathway, thereby enhancing cellular antioxidant capacity. Nrf2 activation upregulated downstream cytoprotective enzymes heme oxygenase-1 (HO-1) and NAD(P)H quinone dehydrogenase 1 (NQO1), reduced reactive oxygen species (ROS) accumulation, and diminished the release of transforming growth factor-β1 (TGF-β1), effectively mitigating oxidative stress. Notably, the antioxidant action of PD was markedly attenuated in Nrf2-deficient cellular models, underscoring the centrality of Nrf2 signaling to PD’s mechanism (Huang et al., 2015).

#### 4.6.4 Regulation of ferritinophagy and ferroptosis

Ferroptosis and ferritinophagy are critical regulatory nodes in asthma progression, and their aberrant activation is closely associated with disease exacerbation (Li et al., 2023). In an ovalbumin (OVA)-induced rat model, Li et al. (2025) showed that PD suppresses NCOA4-mediated ferritinophagy, thereby limiting Fe^2+^ release and preventing ferroptotic injury in lung tissue; PD also reduced intrapulmonary ferritin deposition. Through these coordinated actions, PD significantly attenuated airway inflammation, improved pulmonary function, and alleviated airway remodeling, yielding meaningful disease modification.

Mast cells are pivotal in asthma pathobiology and constitute an important therapeutic target (Joulia et al., 2024). *In vivo*, Yuan et al. (2012) found that PD downregulated UCN expression, blocked Ca^2+^ influx, and inhibited mast-cell degranulation—mechanisms that further expand the spectrum of PD’s anti-asthmatic activities.

**FIGURE 7:**
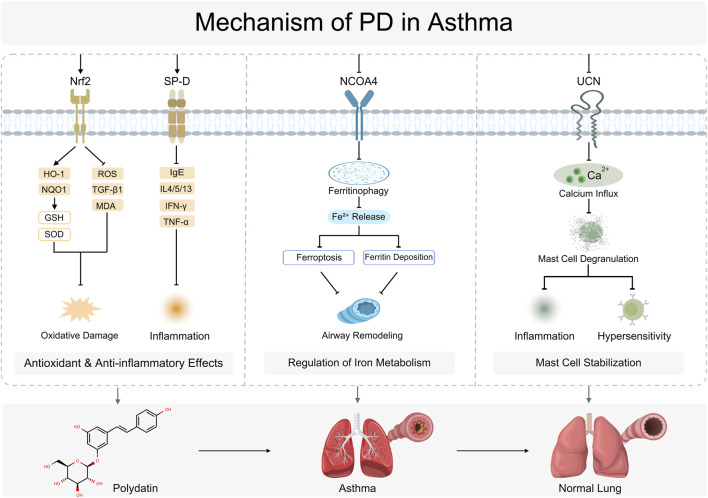
Mechanism of PD in Lung Cancer. NOD-, LRR- and pyrin domain-containing protein 3 (NLRP3), Nuclear factor kappa-light-chain-enhancer of activated B cells (NF-κB), Tumor necrosis factor alpha (TNF-α), Interleukin-1 beta (IL-1β), Cholangiocarcinoma (CCA), BCL2-associated X protein (Bax), B-cell lymphoma 2 (Bcl-2), Caspase-9 (CASP9), Caspase-3 (CASP3), Epidermal growth factor receptor (EGFR), Pembrolizumab plus Cisplatin (PD + Cisplatin), NADPH oxidase 6 (NOX6), Reactive oxygen species (ROS), Estrogen receptor (ESR), c-Jun N-terminal kinase (JNK), p38 mitogen-activated protein kinase (p38 MAPK), B-cell infiltration (B-cell infiltr.), Pembrolizumab plus Afatinib (PD + Afatinib), Epidermal growth factor receptor modulation (EGFR Mod.), Local Anti-inflammatory (Local Anti-inflamm.), Inflammation (Inflamm.).

In sum, PD exerts clear therapeutic effects in asthma *via* multidimensional and synergistic mechanisms. It activates the Nrf2/HO-1 axis to curb oxidative stress, lowers pro-inflammatory cytokine levels, and suppresses UCN expression and mast-cell degranulation to relieve airway inflammation. In parallel, PD modulates ferritinophagy and ferroptosis to correct iron-handling abnormalities, thereby further limiting airway remodeling and improving lung function. These integrated actions highlight PD’s unique advantages and translational potential in the treatment of asthma (Figure 7).

## 5 Conclusions and future perspectives

Respiratory diseases rank among the leading global causes of morbidity and mortality, posing a substantial threat to human health and an immense burden on public-health systems. As a natural polyphenolic compound, polydatin (PD) has demonstrated notable pharmacological activity and therapeutic potential across multiple respiratory disorders. This review synthesizes current evidence on PD in pulmonary fibrosis, acute lung injury/acute respiratory distress syndrome (ALI/ARDS), pneumonia, lung cancer, and asthma, encompassing anti-inflammatory actions, attenuation of oxidative stress, inhibition of epithelial-mesenchymal transition (EMT), modulation of the gut microbiota, reprogramming of the tumor microenvironment, and regulation of apoptosis and autophagy. At the molecular level, PD engages key signaling pathways—including NF-κB, NLRP3, TGF-β/Smad, PI3K/AKT/mTOR, and Nrf2/HO-1—to coordinately regulate inflammatory networks, redox homeostasis, and cell fate, thereby mitigating pathological injury and improving disease trajectories. Notably, in preclinical models of pulmonary fibrosis, pneumonia, and asthma, PD has yielded efficacy comparable to—or in certain settings exceeding—that of standard agents in those specific experimental contexts; in lung cancer combination regimens, PD has further exhibited chemosensitizing/radiosensitizing effects with toxicity-sparing benefits *in vitro* and in animal studies. Collectively, these studies delineate PD’s multidimensional mechanisms, therapeutic advantages, and potential clinical utility in respiratory medicine, providing a strong conceptual and preclinical foundation for deeper mechanistic interrogation and translational advancement. Nevertheless, important limitations continue to hinder clinical adoption, and substantial challenges must be overcome before PD can be widely implemented in practice.

### 5.1 Deep mechanistic dissection and target identification

Although numerous studies implicate PD in anti-fibrotic, anti-inflammatory, antioxidant, and antitumor effects *via* modulation of multiple pathways, much of the literature remains at the levels of phenotypic observation and pathway validation. The direct molecular targets of PD have not been unequivocally identified, and it remains unclear whether one or several upstream nodes coordinate the observed downstream effects (e.g., inhibition of NF-κB, activation of Nrf2, regulation of NLRP3) or how these pathways interrelate. To address this gap, we propose an integrated “target screening-validation-mechanism confirmation” strategy:

Chemobiology-based capture of candidate binders: Employ affinity pulldown using PD-based probes to capture putative binding proteins from lung tissue or relevant cell systems, and complement this with drug-affinity responsive target stability (DARTS) assays to prioritize high-confidence candidates based on protease-resistance shifts.

Systems pharmacology and quantitative proteomics: Construct a “PD-disease-target” regulatory network by integrating multisource databases to infer key nodes; then apply quantitative proteomics to map PD-induced dynamics in candidate targets and their interactors (expression and post-translational modifications), thereby functionally validating target involvement within the pertinent pathways.

Binding-mode definition and biophysical confirmation: Perform molecular docking to model PD-target interactions and experimentally determine binding affinities, enabling definitive identification of direct targets and clarification of their mechanistic relevance.

### 5.2 Formulation optimization and precision delivery

PD’s poor aqueous solubility, low bioavailability, and complex *in vivo* disposition—together with limited data on pulmonary enrichment, residence time, and cellular targeting—constrain translational progress. Although co-crystals (e.g., PD-L-proline) and nano-delivery systems (e.g., hyaluronic acid/lactoferrin-coated PLGA nanoparticles) have improved solubility and targeting in preclinical settings, human pharmacokinetic data are lacking. Notably, the doses of PD employed across preclinical studies vary considerably, with *in vivo* experiments typically using 10–200 mg/kg *via* oral or intraperitoneal routes and *in vitro* studies relying on concentrations ranging from low to high micromolar levels. This heterogeneity, while reflecting differences in disease models, treatment schedules, and experimental objectives, complicates the extrapolation of these regimens to clinically feasible dosing strategies and underscores the need for systematic pharmacokinetic bridging. To advance the clinical translation and therapeutic potential of this research, future efforts should prioritize several key avenues. First, the development of lung-targeted delivery platforms—such as inhalable nanomedicines, exosome-based carriers, and liposomal systems—is crucial to extend pulmonary drug residence and enhance site-specific targeting. Second, strategies that combine rational prodrug design with biomaterial encapsulation should be employed to systematically improve drug solubility, metabolic stability, and bioavailability. Concurrently, integrated pharmacokinetic/pharmacodynamic (PK/PD) modeling must be conducted to quantitatively delineate the *in vivo* disposition and effect relationships of the therapeutic agents, thereby providing a sound basis for the rational design and optimization of clinical dosing regimens. The concerted progress along these research fronts will lay a critical foundation for the development of more effective and precise therapeutics for respiratory diseases.

### 5.3 Strengthening clinical research and building an evidence base

Current investigations are dominated by cell and animal studies, with a paucity of randomized clinical trials and real-world evidence (RWE). While randomized controlled trials (RCTs) of PD-containing preparations have reported benefits in neurological and gastrointestinal indications (e.g., brain tumors, irritable bowel syndrome), these findings cannot be directly extrapolated to respiratory diseases. To date, only preliminary work by Fuggetta and colleagues has explored prophylactic PD cream for afatinib-associated skin toxicity; however, that study enrolled just 34 lung-cancer patients, lacked a control arm, and involved short follow-up, limiting the strength of evidence. Additional heterogeneity across preclinical models—such as species and induction methods in pulmonary fibrosis—and wide dose ranges (e.g., ∼20–160 mg/kg) further constrain comparability and generalizability. We recommend a staged clinical program:

Phase I trials: Evaluate safety, tolerability, and human PK profiles of PD.

Disease-specific Phase II/III RCTs: Conduct large, multicenter trials in pulmonary fibrosis, lung cancer, pneumonia, and other respiratory conditions to establish long-term efficacy and safety across diverse populations; benchmark PD—alone or in combination—against standard-of-care regimens to define comparative advantages.

Dose optimization: Integrate Phase II/III results with PK/PD analyses to refine dosage and scheduling and to develop standardized clinical protocols.

RWE studies: Leverage electronic health records and payer/claims databases to address questions not fully captured in RCTs—long-term effectiveness, special populations, rare adverse events, and cost-effectiveness—thereby complementing the totality of evidence.

### 5.4 Developing combination-therapy strategies

As a natural compound, PD shows promise for enhancing efficacy, reversing resistance, and attenuating toxicity when combined with standard therapies. Although synergistic effects have been reported *in vitro* with cisplatin and afatinib, the current evidence base is limited: studies are few, mechanistic chains are incomplete, and most data are confined to cell systems without robust *in vivo* or clinical corroboration. To systematically advance combination strategies, we propose:

A structured combination-screening framework: Curate a library of “PD-standard therapy” pairs with putative mechanistic complementarity for respiratory diseases; apply high-throughput screening integrated with network pharmacology and multi-omics to delineate interaction mechanisms and nominate rational combinations.

*In vivo* efficacy and safety evaluation: Use human respiratory organoids and/or pathophysiologically relevant animal models to assess antitumor/anti-fibrotic/anti-infective efficacy, PK characteristics, and safety endpoints; iteratively optimize dosing regimens.

Clinical translation: Progress stepwise to clinical trials to establish efficacy, tolerability, and safety of PD-based combinations in humans.

## 6 Final synthesis and translational outlook

Overall perspective: PD exhibits multi-dimensional utility across pulmonary disorders and holds considerable translational promise. Realizing its clinical potential will require coordinated advances in mechanistic elucidation, formulation science and delivery, and rigorous clinical evaluation. With continued methodological innovation and sustained clinical research, the current challenges are likely to be surmounted, enabling PD to play an increasingly substantive role in the prevention and treatment of respiratory diseases (Table 1).

**TABLE 1:** Effects of polydatin in respiratory diseases.

Disease	Models and modeling methods	Research type	Dose and treatment schedule	Targets	Comparator drug	Effects	References
Pulmonary fibrosis	SD rats; BLM(5 mg/kg) endotracheal in a single drug delivery; HFL-1 cells; TGF-β(10 ng/mL) induce 48 h	*In vivo* and *in vitro*	PD:100 mg/kg/d,i.p. for 28days; PD:50,150uM for 48 h	↓TGF-β1,↓collagen I,↓collagen III in the lung tissue; ↓TNF-a,↓IL-1β,↓IL-6,↓IL-17,↓PICP、↓PIIINP in serum; ↑E-cadherin,↓fibronectin, ↓TGF-β, ↓p-Smad2/Smad2,↓p-Smad3/Smad3 in cells	—	Suppressing inflammation and EMT *via* inhibiting TGF-β/Smad signaling pathway	Qiu et al. (2019)
	MRC-5 cells; PQ (100 μmol/L) induce 24 h	*In vitro*	PD:60,80,100uM,treated for 24 h	↓GSH-Px,↓MDA,↓SOD,↓TNF-α,↓TGF-β,↓L-1β,↓IL-6,↓NLRP3,↓caspase-1,↓ASC	—	Reducing the inflammatory response, improving the antioxidant stress capacity, and inhibiting the activation of the NLRP3 inflammasome	Fu et al. (2020)
	C57BL/6 mice; BLM(5 mg/kg),intratracheal in a single drug delivery	*In vivo*	PD:40 mg/kg/d,i.g. for 28 d	↓Collagen I,↓α-SMA in the lung tissue,↓TNF-α,↓LPS,↓IL-6,↓IL-1β in serum; ↑Muribaculaceae, ↑*Lactobacillus*, ↑Akkermansia in the intestines	—	Reducing the inflammatory response, altering gut microbiota species abundance	Yang et al. (2023)
	SD rats; MTX (14 mg/kg),a single oral dose per week for 2w	*In vivo*	PD:25,50,100 mg/kg/d,p.o. for 14 d	↑GSH,↓TGF-β,↓α -SMA,↓MDA,↓IL-1β,↓HYP↓,TNF-α,↓8-OHdG) in serum	—	Anti-oxidant, anti-inflammatory as well as anti-fibrotic	Ali et al. (2022)
	SD rats; BLM(5 mg/kg),intratracheal in a single drug delivery; A549 cells; TGF-β(10 ng/mL) induce 96 h	*In vivo* and *in vitro*	PD:10,40,160 mg/kg/d,i.g. for 28days; PD:10,30,90 uM for 96 h	↓α-SMA,↓Collagen I,↑E-cadherin,↓MDA,↓MPO,↓TNF-α,↓IL-6,↓IL-13,↑SOD,↓TGF-β1,↓p-Smad2/3,↓p-ERK1/2 in the lung tissue; ↓α-SMA/Collagen I,↑E-cadherin,↓EMT in A549 cells	Pirfenidone, (50 mg/kg)	Relieving oxidative and inflammatory stress and inhibition of EMT and collagen deposition regulated by Smad-dependent and Smad-independent TGF-ff signals	Liu et al. (2020)
	BALB/c mice; MP infection + PD BEAS-2B cell; MP infection + PD	*In vivo* and *in vitro*	MP(50 μL 1 × 10^8^TU/mL)+PD (100 mg/kg) 7 days PD (100 μM 2 h)+MP(1 × 10^6^TU/mL 24 h)	↓IL-6,↓IL-1β,↓TNF-α,↓MCP-1; ↓TGF-β,↓α-SMA,↓Type I collagen,↓Type III collagen	—	Suppressed the inflammatory response and the development of pulmonary fibrosis by inhibiting the NLRP3 inflammasome and NF-κB pathway	Tang et al. (2019)
	C57BL/6 mice; BLM(5 mg/kg),intratracheal in a single drug delivery	*In vivo*	Jianghu decoction (JH), (PD) was identified as the active ingredient in JH	↓HMGB1,↓RAGE,↓SEPTIN4,↓ACTA2, ↓ITGAV,↑AMPK/PGC1α/PPARγ,↓AMPK/HMGB1/RAGE	—	Exerting synergistic inhibition on pulmonary fibrosis and inflammation	Zhang et al. (2024a)
	BEAS-2B and 16HBE cells; radon gas (Ra source) at 20,000 Bq/m^3^ for 20 min each time, once every 3 days, for a total of 6 times (Rn6 cells) Mice; inhaled 105 Bq/m3 radon gas,120WLM	*In vitro*	100,200 μM for 24 h; 200 μM before and after exposure; PD:50,100 mg/kg/d,i.p.before and after exposure	↓ROS,↑SOD,↑E-cad,↓Vimentin/FN1/N-cad/α-SMA/Snail,↓p-AKT,↓p-mTOR in cells; ↑SOD,↓MDA in serum; ↓FN1/α-SMA/Vimentin/Snail,↓p-PI3K,↓p-AKT,↓p-mTOR in the lung tissue	—	Reducing oxidative stress, weakening epithelial-mesenchymal transition EMT and lung fibrosis *via* inhibiting the PI3K/AKT/mTOR pathway	Chen et al. (2024)
	SD rats; 1 mL of silica suspension by the non-tracheal exposure method	*In vivo*	PD:30 mg/kg/d,i.g. for 4w,8w	↓CVF,↓HYP,↓TNF-α,↓IL-6,↓IL-1β,↓p53,↓Caspase3 in the lung tissue; ↓p_Elusimicrobiota,↓g_Acidibacter,↑SCFAs,↑propionate,↑pelargonic acid,↑acetate in the intestines	—	Inhibiting the expression of inflammation-related indexes and aptosis-related indexes, regulating the diversity of the intestinal flora and tcontent of short-chain fatty acids	Wu et al. (2025)
	SD rats; aPM2.5 (47.1 mg/m^3^), exposed 60 min/day for 4w,8w	*In vivo*	PD:50 mg/kg/d,i.g. for 4w,8w	↓OP,↓ROS,↑Nrf-2,↑PPAR-γ,↓TNF-α、↓IL-1β,↓Cer 18_0_1P,↓dhCer 18_0_1P in the lung tissue; ↓MDA,↑GSH-Px,↓WBC,↓LYM,↓MON in the BALF	—	Prevented the lung function decline, reducing the level of oxidative, Inhibiting inflammation response	Yan et al. (2017)
	BALB/c mice; BLM(50 μL,1 mg/mL),intratracheal in a single drug delivery	*In vivo*	PD + Cur:14.92,7.46,3.73 mg/kg i.p. qod for 4w	↓MUC5B,↓KL-6,↓SP-D,↑TOLLIP,↑RCN3,↓IL-6,↓CCL18,↓SF in serum; ↓PI3K/AKT/TGF-β,↓PI3KR1,↓AKT,↓TGFβR3 in the lung tissue	Nintedanib (17.1 mg/kg i.p. Qod for 4w	Activating GABBR, inhibiting the PI3K/AKT/TGF-βpathway, alleviating immune inflammatory response and pulmonary fibrosis	Zhen et al. (2025)
	BALB/c rats OVA-induced asthma; BEAS-2B Cells TGF-β1-induced EMT; BEAS-2B Cell Nrf2-knockdown	*In vivo* and *in vitro*	OVA 7 days + 100 mg/kg PD; 100 μM PD +10 ng/mL TGF-β1 48h; 50 nM Nrf2 siRNA (siNrf2)transfected	↓ROS,↓TGF-β1,↓fibroblasts,↓EMT in the lung itssue; ↑Nrf2,↓NOX1/4,↑HO1,↑NQO1,↓vimentin,↓α-SMA↓,Type I collagen, ↓Type III collagenin cells	—	Inhibiting activity of ROS and TGF-β1,suppressing EMT and lung fibroblast protein expression in lung tissue	Zeng et al. (2019)
ALI/ARDS	C57BL/6 mice; cecum ligation and puncture (CLP); PMVECs; exposed to LPS (100 ng/mL) for 24 h at 37 °C	*In vivo* and *in vitro*	PD:50 mg/kg I.g. were performed 48 h before CLP; PD:0.5 mM Incubate for 4 h before LPS	↓Spi-B, ↓p-PI3K, ↓p-Akt, ↓p-NF-κB1 in lung tissue and PMVECs; ↓IL-6, ↓TNF-α, ↓IL-1β, ↓IFN-α in BALF and PMVECs supernatant	Dex:40 mg/kg I.g.	Alleviating inflammatory responses, downregulating Spi-B expression, and suppressing the activation of PI3K/Akt and NF-κB signaling pathways	Liao et al. (2023)
	SD rats; LPS(5 mg/kg)endotracheal in a single drug delivery	*In vivo*	PD:15,30,45 mg/kg i.v. for 30 min,then LPS injection 24 h	↓TNF-α, ↓IL-1β, ↓IL-6, ↓HMGB1, ↓LPO in serum; ↓MPO,↓caspase 3, ↓Bax, ↑Bcl-2 in lung tissue	—	Reducing lung inflammation, inhibiting lung cell apoptosis, and alleviating oxidative stress	Li et al. (2014a)
	C57BL/6 mice; 60Co source local thoracic irradiation (single dose 15 Gy, dose rate 1 Gy/min); BEAS-2B cells; 8 Gy irradiation (dose rate 2 Gy/min)	*In vivo* and *in vitro*	PD:100 mg/kg/d i.p. before and after irradiation; PD: Unspecified concentration, pretreated BEAS-2B cells 1 h prior to irradiation,	↑Sirt3, ↑Nrf2, ↑PGC1α,↓TGF-β1, ↓p-Smad3,↓Snail,↓MDA,↑SOD in lung tissues; ↓IL-4, ↓IL-13, ↓TNF-α, ↓ET-1, ↓PGE2 in serum; ↑E-cadherin, ↓Vimentin, ↓α-SMA in lung tissues and cells	—	Scavenging free radicals, inhibiting EMT and TGF-β1-Smad3 signaling, reversing Th1/Th2 imbalance, and activating Sirt3, Nrf2 and PGC1α	Cao et al. (2017)
	Mice (6-week-old); radon exposure (10^5^ Bq/m^3^, 10 h/d, 6 days/w, totaling 755 h; BEAS-2B and 16HBE; Radon exposure (every 3 days, up to 6th passage) | Rn6 cells utilized	*In vivo* and *in vitro*	PD:50,100 mg/kg, i.p. for 30min before exposure; PD: 100, 200 μmol 24 h after exposure; PD:200μmol, added before and after exposure	↑E-cad, ↓FN1, ↓Vimentin, ↓N-cad, ↓α-SMA, and ↓Snail,↓ p-PI3K, ↓p-AKT, and ↓p-mTOR in cells; ↑SOD,↓ MDA incells and serum	—	Reducing oxidative stress, weakening EMT and lung fibrosis, inhibiting PI3K/AKT/mTOR pathway	Chen et al. (2023)
	SPF SD rats; Fluid percussion injury (FPI): Craniotomy (2.5 mm lateral to sagittal sinus),then Epidural cannula fixation and Saline pulse (3.5 ± 0.2 atm, 21–23 ms)	*In vivo*	PD:30 mg/kg,i.p. after FPI	↓MDA,↑ SOD,↓IL-6, ↓IL-1β, ↓TNF-α, ↓MCP-1,↓S100B,↓MPO, ↓NE, ↓CitH3 in lung tissue	—	Inhibiting the expression of S100Band NETs formation, promotting permeability recovery, alleviating oxidative stress response and inflammatory cytokines release	Gu et al. (2021)
	RAW 264.7,HEK 293T; LPS(1 μg/mL) induce 12h; KM mice; activated *Pseudomonas aeruginosa* PA-14 strain (PBS,2 × 10^6^ CFU/20 μL) infect Nasal cavity	*In vivo* and *in vitro*	RES:0.05,0.5,5 μM incubate 12 h following LPS stimulation; RES:20,40,80 mg/kg i.p. and RES probe 280 mg/kg for 24 h	↓KEAP1-NRF2,↑NRF2,↑HO1,↑GCLC,↓ROS in cells and lung tissue; ↓TNF-α,↓IL-1β,↓IL-6 in serum	—	Disrupting the binding between KEAP1 and NRF2, activating NRF2 upregulation of nuclear transcription, enhancing the expression of anti- oxidant genes dependent on ARE, suppresses ROS generation	Chi et al. (2024)
	SD rats; exposing the shaved dorsa area (30%) to 98 °C water for 30s,then using Ringer’s lactate solution (4 mL/kg per percentage of the burn) i.p.	*In vivo*	PD:45 mg/kg,i.v. for 24 h or observing survival time	↑Bax,↑Bcl-xl,↓caspase-3, ↓MPO in lung tissue; ↓TNF-α,↓IL-1β.↓IL-6 in lung tissue and serum	—	Inhibiting inflammatory response and apoptosis, reducing permeability of pulmonary microvessels	Li et al. (2014b)
	C57BL/6 mice and Parkin^−/−^ mice; LPS(5 mg/kg) endotracheal in a single drug delivery; BEAS 2B cells; LPS(0.5 mM) induce 6 h	*In vivo* and *in vitro*	PD:45 mg/kg,i.v. for 12h; PD:50 μM incubate for 6 h following LPS stimulation	↓cycs in cytoplasmic; ↑Parkin in mitochondria; ↓Bax,↑Bcl-2,↓caspase-3 in cells; ↓LIS,↓apoptotic cells count in the lung tissue	—	Activating Parkin-dependent mitophagy, inhibiting mitochondrial-dependent apoptosis	Li et al. (2019)
	SD rats; LPS(10 mg/kg) right jugular vein injection; BEAS-2B cells; LPS(100 ng/mL) induce 24 h	*In vivo* and *in vitro*	PD:1,5,10,30mg/kgwas intraperitoneall injected 1 h later or 0.5 h before LPS,and incubate to 6 h after LPS given; PD:0.5 mM incubate when LPS have been given 4 h or 24 h,contnue to incubate to total duration 28 h	↓sPLA2,↓cPLA_2_mRNA, ↑Clara cell,↑CCSP in the lung tissue,↑CCSP in cells and serum	—	Upregulating the mRNA and protein expression of CCSP, inhibiting the expression of sPLA_2_ and cPLA_2_, decreasing serum CCSP to reduce the permeability of the lung - blood barrier	Shu et al. (2011)
	50 BalB/c mice; LPS(8 mg/kg)endotracheal in a single drug delivery; BEAS-2B cells; LSP(2 μg/mL) induce 20 h or 24 h	*In vivo* and *in vitro*	PD:20,80 mg/kg was intraperitoneall injected 1 h before LPS,and incubate to 6 h after LPS given; PD:2,4, 8 μM incubate 2 h before LPS,and incubate to 20 h or 24 h after LPS given	↓MPO,↓TLR4,↓MyD88,↓IRAK1,↓IKKα,p-IKKα,↓IKKβ,p-IKKβ,↓IκBα,p-IκBα,↓NF-κB,↓TNF-α,↓IL-6,↓IL-1β in lung tissue; ↓IL-1β,↓IL-6,↓IL-8,↓TNF-α,↓TLR4,↓MyD88,↓NF-κB in cells	DEX:5 mg/kg i.p.	Inhibiting the TLR4-MyD88-NF-κB signaling pathway, reducing the release of inflammatory factors and neutrophil infiltration	Jiang et al. (2015)
pneumonia	Vero E6 and MDCK cells; A/Puerto Rico/8/34 H1N1 (PR8), SARS-CoV-2 virus infection, cell monolayers with the virus for 1 h, 2% FCS 24 h	*In vitro*	PD/A5+: 5–20 μg/mL (H1N1), 10–40 μg/mL (SARS-CoV-2); added for 24 h	PD:↓NP, ↓HA; A5+: ↓NP/HA,↓IL-6 in Vero E6 cells; SARS-CoV-2: ↓TCD50, ↓S/N in MDCK cells	—	Suppressing viral HA, NP protein expressions and viral titer, reducing IL-6 secretion in influenza A virus-infected cells, alleviating virus-induced inflammatory response	Ogando et al. (2020)
	BEAS-2B cells; MP(1 × 10^7^ CCU/m) for 24 h; BALB/c mice; intranasal administration of MP (1 × 10^7^ CCU/mL) at a volume of 50 μL, twice a day for 5 d	*In vitro* and *in vivo*	PD:24 h after MP infection; PD:100 mg/kg of PD for 5 d	↑Na,K-ATPase activity,↓caspase-1/GSDMD/GSDMD-N,↓IL-1β/IL-18 in BEAS-2B cell; ↓LDH,↓caspase-1/GSDMD/GSDMD-N,↓ IL-1β/IL-18,↑Na,K-ATPase activity in lung tissue	—	Suppressing the caspase-1/GSDMD-dependent pyroptosis pathway with dose-dependent effects, alleviating MP-induced lung injury and systemic inflammation	Liping et al. (2020)
	RAW 264.7 cell; treated LTA or in combination PD;	*In vitro*	PD:25,50,100 mg/kg,once every 8 h for a total of 3 times; PD:50 l g/mL for 1h, and then, LTA (5 l g/mL) added for 3 h	↓ROS,↓TLR2 mRNA,↓p-p65,↓p-IκBα,↓NF-κB p65,↓TNF-α,↓IL-1β,↓IL-6 mRNA,↓caspase 9/3 in cell	LAT	Alleviated uterine pathological damage, reduced ROS production in macrophages, inhibited TLR2-NF-κB pathway activation, decreased pro-inflammatory cytokine expression and cell apoptosis.	Sinha et al. (2005)
	Recombinant Mpro + fluorescent peptide substrate QS1	*In Vitro*	PD:100 μM incubated with 100 μM Mpro at 37 °C for 10min, then 50 μM QS1 added, detected at 37 °C for 30 min	↑Mpro enzyme activity inhibition rate,↑Fluorescence Intensity	N3	Binding to the catalytic residues (Cys145, His41) and key residues of Mpro, thereby inhibiting the function of Mpro in cleaving viral polyproteins and further blocking the replication of SARS-CoV-2	Wu et al. (2020)
	Calu-3/A549/BEAS2B/16HBE (NSP6 over expression); 157 COVID-19 sera +52 controls	*In Vitro*	PD: Transfecting NSP6 plasmids into lung epithelial cells (Calu-3/A549/BEAS2B), detect indicators after 24 h co-culture	↑LC3B-II/p62, ↓LysoTracker Red; ↑caspase-1/GSDMD-NT/IL-1β/IL-18; ↑M65 all from Calu-3/A549/BEAS2B/16HBE	Autophagy/lysosome Mild-moderate COVID-19	Restoring lysosomal acidification,relieving autophagic flux stagnation,inhibiting NLRP3 inflammasome,alleviates lung pyroptosis	Wang et al. (2020)
	BALB/c mice; MPP and MPP + PD groups inoculated with 50 μL of MP (∼1 × 10^8^ TU/mL); Cells: BEAS-2B cells; PD-pretreated groups incubated with 100 μM PD for 2 h, MP-stimulated groups MP (1 × 10^6^ TU/mL) 24 h	*In vivo* and *in vitro*	PD:100 mg/kg at 2 h pre-MP; PD:100 μM at 2 h pre-MP	↓IL-6, ↓IL-1β, ↓TNF-α, ↓MCP-1,↓Lung epithelial cell apoptosis, ↓Caspase-3,↓NLRP3, ↓NF-κB p65 in lung tissue and serum; consistent with up in BALB/c mice cell	—	Reducing serum inflammatory factor levels, decreasing pulmonary epithelial cell apoptosis, downregulating the expression of fibrosis-related proteins	Wang et al. (2018)
	RAW264.7, HEK 293T cells; LPS-induced oxidative injury KM mice; activated *Pseudomonas aeruginosa* PA-14 strain (2 × 10^6^/20 μL in PBS)	*In vitro* and *in vivo*	PD:0.05,0.5,5 μM for 12 h	↓ROS, ↓KEAP1, ↑NRF2/GCLC/ARE; ↓TNF-α,↓IL-1β,↓IL-6↓in RAW cell; consistent with up in lung tissue	RES	Reducing ROS production, activates the NRF2/ARE pathway and upregulates antioxidant proteins, exerting pulmonary oxidative stress relief and anti-inflammatory effects *via* *in vivo* conversion to RES	Baird et al. (2020)
	RD Cell-OC43 infected with HCoV-OC43; 293T-hACE2 cells; Recombinant 3CLpro/PLpro of SARS-CoV-2/SARS-CoV	*In vitro*	PD:1.234μM, 3.7μM, 11.11μM, 33.33μM, 100 μM for 48 h at 37 °C; 3CLpro/PLpro (1 µM final concentration) at 4 °C for 2 h;	↓N protein in RD cell,↑CC_50_ in 293T-hACE2 Cell; ↓PLpro enzyme activity,↓Binding affinity in Recombinant	DMSO	Inhibiting HCoV-OC43 replication, inhibiting 3CLpro and PLpro activities	Rozdzal et al. (2020)
	Chicken embryo fibroblast cell line; 0.2 mL MG-HS (1 × 10^9^ CCU/mL) for 6 h; 90 specific pathogen-free (SPF) fertilized chicken eggs; 0.8 mL MG-HS (1 × 10^9^ CCU/mL)	*In vivo* and *in vitro*	PD:15, 30, 45 mg/kg at 3, 5, 7; PD: 15, 30, 60 μg/mL for 48 h	↓MG growth,↑Cell viability,↓G1 phase ratio,↓Apoptosis rate,↓TLR6,↓MyD88,↓NF-κB,↓NF-κB-p65,↓IL-1β,↓IL-6,TNF-α in DF-1 cells; ↓TLR6,↓MyD88,↓NF-κB,↓IL-1β,↓IL-6,↓TNF-α in the lung tissue	Tylosin	Inhibiting the TLR6/MyD88/NF-κB pathway, alleviating MG-induced lung inflammatory lesions	Jiang et al. (2015)
Lung cancer	LLC cell; 10% fetal bovine serum and 1% penicillin/streptomycin in a 37 °C, 5% CO_2_ incubator; mice; inject 1 × 10+LLC cells	*In vitro* and *in vivo*	PD: 30 μmol for 48 h PD: 10,20,30 mg/kg for once every 2 d	↓TGF-β1,↓collagen I,↓collagen III in the lung tissue; ↓TNF-a,↓IL-1β,↓IL-6,↓IL-17,↓PICP,↓PIIINP in serum; ↓TGF-β in cells	—	Inhibiting the invasion (migration) and proliferation of NSCLC cells, inhibiting NSCLC tumor growth	Fan and Shuai (2024)
	People with NSCLC: Age ≥18 years, ECOG physical status 0–2 points, common activation mutations of EGFR, receiving first-line afatinib therapy	*In vitro*	1.5% PD-based cream: 2 times a day starting 1 day before afatinib treatment and continuing until the end of afatinib treatment	Keratinocyte-interleukin-8↓	—	Reducing the incidence of skin toxicity	Fuggetta et al. (2024)
	Human lung cancer (A549) cell line; maintained as monolayer culture (2 D); with 10% fetal bovine serum (FBS) and 1% antibiotics solution,at 37 °C with 5% CO_2_	*In vitro*	PD:0–1 mM for 72 h	↑SA-b-galactosidase at 0.2–0.6 mM,↑cH2AX,↑SA-b-galactosidase at 0.6–0.8 mM,and ↓Ki67,↑cH2AX,↓procaspase-3/9,↑SA-b-galactosidase,↑p21,↑p53 at 0.8–1 mM in cells	Everolimus	Inducing DNA damage in a concentration dependent manner, inducting the cells enescence/apoptosis	Verma et al. (2020)
	Mice; inject 549-lucif-erase BALB/c mice; Co-γ-ray radiotherapy for 8, 9, and 10 Gy (dose rate 1 Gy/min); cells:monitories tumor volume every 3 days, PD and IR + PD groups with the PD (20 mg/mL, 200 μL), 7 days,A549 cells; DMEM medium	*In vivo* and *in vitro*	PD:20 mg/mL, 200 μL 7 days; PD:0, 20, 40, 60, 80, 100, 120 μM (CCK-8); 50 μM, 100 μM added 1 h before radiotherapy, cultured 24/48/72 h after radiotherapy (CCK-8), 12 days (clonogenesis), 48 h (apoptosis)	↓B220,↓CD19 in the lung tissue; ↓A549 in cells	DMSO	Inhibiting radiotherapy-induced tumor-infiltrating B cells, reducing normal tissue damage from systemic radiotherapy	Guo et al. (2023)
	Lung cancer (A549,NCI-H1975); with 10% fetal calf serum, 2 mmol/L glutamine,100 μg/mL streptomycin and 100 U/mL penicillin	*In vitro*	PD:2,4,6 μmol/L for 20 h,44 h,68 h	↓Bcl-2,↑Bax,↓Cyclin D1,↑Bax/Bcl-2 in cells	DMSO	Broading spectrum inhibition of cancer cell proliferation; dose-dependent inducting the lung cancer cell apoptosis; inducing S-phase cycle arrest in lung cancer cells	Zhang et al. (2014)
	NSCLC cell lines (A549, H129); with 10% FBS at 37 °C with 5% CO_2_	*In vitro*	PD:25,50,100 μM for 0,1,2,3 d	↓NI.RP3, ↓ASC, ↓pro-caspase-1, ↓caspase-1,↓phosphor-NF-κB p65 in cells	ATP: 25 μM TNF-α:20 ng/mL Mitomycin C:10 μg/mL	Inhibiting NLRP3 inflammasome activation, inhibiting NSCLC cell migration directly	Zou et al. (2018)
	Cells: H1299, H460, BEAS-2B in RPMI-1640 medium MIHA, HUVEC in F-12K medium mice: With stable RT and humidity (50%–60%),a 12-h light/dark cycle, inject25 × 106 H460 cells per 100 μL of PBS/Matrigel mixture (1:1), (*n* = 5),with cisplatin or vehicle	*In vivo* and *in vitro*	PD:0.200,300,400,500,600 μM for 8 h,15 h,72 h; PD:50 mg/kg once every 2 days for 2w	↓H1299,↓H460,↑Cleaved-Caspase3,↓Bcl-2 in cells; ↑NOX5,↑p-eIF2α,↑ATF4,↑p-JNK,↑p-p38,↓Ki-67 in the lung tissue	DMSO, Vehicle,Cisplatin; NAC; NC siRNA; Carboplatin	Activating the NOX5-ROS-ER stress/MAPK pathway to inhibite NSCLC cells and to enhance cisplatin’s antitumor activity	Wu et al. (2024)
	The A549 cancer cell line (murine origin); Culture of 10% fetal bovine serum and 1% penicillin/streptomycin in RPMI 1640 medium; HEK-293 (human origin) cell; DMEM	*In vitro*	PD-L-Pro eutectic:at 60 °C for 30 min, then cooled to 25 °C, and crystallized after 24 h	↓A549 in cells	—	Cytotoxicity against for A549	Lou et al. (2021)
	5 A549cells; Polydatin (PD)/PLGA nanoparticles (F1) were prepared by nano-precipitation method, and targeted nanoparticles (F4, F7, F9, etc.)	*In vitro*	PD:0.001–100 μg/mL for 72 h at cytotoxicity test; 20 μg/mL for 24 h at cell uptake assay	↓VEGF,↓NLRP3, ↓NF-κB in cells	—	Enhancing cytotoxicity and superior cellular uptake efficacy	Nashaat et al. (2024)
Asthma	BALB/c mice; injected 0.2 mL OVA at days 0,7,14,exposing to 1% OVA aerosol 1 h a day for 7days; BEAS2b cell; 10 ng/mL TGF-β1	*In vivo* and *in vitro*	PD:100 mg/kg at days 0,7,14 before OVA stimulation BEAS2b cell:100 μM PD for up to 48 h	↓ROS, ↓TGF-β1 in lung tissues; ↑Nrfe,↑E-cadherin,↓vimentin,↓collagen I, ↓collagen III, ↓α-SMA.,↓NOX1,↓NOX4,↑HO-1,↑NQO1 in cell	—	Promoting Nrf2-mediated antioxidation, inhibiting activity of ROS and TGF-β1	Zeng et al. (2019)
	SD rats; injecting 1% ovalbumin (OVA) sensitization solution on day 1 and day 8,nebulizating with a 1% OVA solution that conducted daily for 4 weeks, with each session lasting 30 min	*In vivo*	PD:200 mg/kg/d for 4w	↓NCOA4,↓Beclin1,↓ Fe^2+^,↓MDA, ↓4-↑HNE,↑FTH1, ↑P62,↑GSH,↑GPX4,↑SLC7A11,↓Fe^3+^ in lung tissues	3-MA,Fer-1	Reducing Fe2+ overload by inhibiting the NCOA4-mediated ferroautophagy, inhibiting ferroptosis in the lung tissues	Li et al. (2025)
	Wistar rats; Sensitization injected with 1 mL of OVA/AlOH3 suspension for sensitization at days 1, 2, 3, and 11 challenge phase:Dropwise 5ug/ul ova for 3 d	*In vivo*	PD:200 mg/kg/d orally for 14 d	↓SP-D and UCN expression in lung tissues; ↓IgE in serum; ↓IL-4, ↓IL-13,↓IL-5,↓TNF-α,↓IFN-γ, ↓iNOS,↑SOD,↑GSH,,↑MDA in BALF; ↓AEC in pellets	Dexamethasone:1 mg/kg/day for 14 d	Significantly reducing the inflammatory mediators and restoring the normal values of oxidative and nitrosative stress biomarkers, reducing the expression of surfactant-D and UCN	Da et al. (2019)
	RBL-2H3 cells: Overnight with 1 μg/mL DNP-specific IgE in 96-well plates; BALB/c mice; injected anti-DNP-BSA-specific IgE (0.5 μg) into the mouse ear	*In vivo* and *in vitro*	PD:100 or 300 mg/kg (200 μL of PBS) or 200 μL of PBS for once	↓FcεRI-mediated degranulation,↓Ca2+ mobilization,↓CRAC,↓ROS accumulation in cells	—	Stabilizing mast cells by suppressing FcεRI-induced Ca2+ mobilization	Yuan et al. (2012)
